# Supplementary material for: The Relationship between Sperm Oxidative Stress Alterations and IVF/ICSI Outcomes: A Systematic Review from Nonhuman Mammals
Source: Biology (Basel). 2020 Jul 21;9(7):178. doi: 10.3390/biology9070178 (PMC7408105; doi:10.3390/biology9070178)
Supplement: Supplementary file 1 [file biology-09-00178-s001.pdf]

Table S1. PICOS design structure, including the inclusion and exclusion criteria and the keywords were used for the definition of the search strategy and the eligibility of the study.

Parameter	*Inclusion*	*Exclusion*	*Keywords*
**POPULATION**	- Mammals different than humans.	- Human beings<br>- Studies in other non-mammals	Mammals, animals, Boar, pig, porcine, swine, rat, hamster, mouse, rodent, bovine, bull, heifer, cattle, ovine, stallion, horse, ram, ovine, goat, buck, dog, cat, donkey, ass, deer, ape, gorilla, chimpanzee
**INTERVENTION**	- Induction and screening for sperm damage<br>- In Vitro Fertilization<br>- Intracytoplasmic Sperm Injection	- Natural mating studies<br>- Intrauterine insemination studies.<br>- Studies assessing the concomitant effect of a treatment in cryopreservation or freeze-drying spermatozoa.	Sperm, oxidative, free radicals, oxidative stress, DNA damage, DNA fragmentation, oxidative damage, superoxide, hydrogen peroxide, 8-oxo-2′-deoxiguanosine.<br><br>ICSI, intracytoplasmic sperm injection, IVF, in vitro fertilization, insemination, blastocyst, embryo.
**COMPARISON**	- Treated and non-treated groups (Treatment vs control).	- Studies that do not analyze the effect of the treatment in sperm cells.	
**OUTCOMES**	- Primary outcomes: Fertilization rate and Blastocyst rate<br>- Secondary outcomes: Implantation and live birth.		Fertilization, blastocyst, pregnancy, implantation.
**STUDY DESIGN**	- Classical Article - Clinical Trial<br>- Observational Study - Comparative<br>- Clinical Study - Cross-sectional	- Review article - Meta-analyses<br>- Systematic reviews - Letters<br>- Case reports - Commentary articles	Classical Article, Clinical Study, Clinical Trial, Clinical Trial, Phase I, Clinical Trial, Phase II, Clinical Trial, Phase III, Clinical Trial, Phase IV, Comparative Study, Controlled Clinical Trial, Corrected and Republished Article, English Abstract, Journal Article, Multicenter Study, Observational Study, Randomized Controlled Trial, English.
